# Blood-Based Clinical Biomarkers of Inflammation and Nutrition in Hyperemesis Gravidarum

**DOI:** 10.3390/jcm13237289

**Published:** 2024-11-30

**Authors:** Busra Demir Cendek, Burak Bayraktar, Zeynep Seyhanli, Ezgi Kocyildiz, Hakan Golbasi, Mujde Can Ibanoglu, Yaprak Engin Ustun

**Affiliations:** 1 Department of Obstetrics and Gynecology, Health Sciences University Etlik Zubeyde Hanim Maternity, Teaching and Research Hospital, 06010 Ankara, Turkey; drmujdecan@gmail.com (M.C.I.); ustunyaprak@yahoo.com (Y.E.U.); 2 Department of Obstetrics and Gynecology, Republic of Turkey Ministry of Health Ankara Etlik City Hospital, 06710 Ankara, Turkey; ezgikocyildiz@gmail.com; 3 Department of Obstetrics and Gynecology, Division of Perinatology, Health Sciences University Etlik Zubeyde Hanim Maternity, Teaching and Research Hospital, 06010 Ankara, Turkey; drburakbayraktar@gmail.com (B.B.); drzeynepseyhanli@gmail.com (Z.S.); 4 Department of Obstetrics and Gynecology, Division of Perinatology, Republic of Turkey Ministry of Health Ankara Etlik City Hospital, 06710 Ankara, Turkey; 5 Department of Obstetrics and Gynecology, Division of Perinatology, Bakircay University Cigli Education and Research Hospital, 35620 Izmir, Turkey; drhkngolbasi@gmail.com

**Keywords:** hyperemesis gravidarum, inflammation, nutrition, HALP score, m-HALP score, prognostic nutritional index

## Abstract

**Background**: In this study, the aim was to investigate blood-based clinical biomarkers of inflammation and nutrition indices in hyperemesis gravidarum (HG). **Methods**: This retrospective case–control study was conducted at a tertiary hospital between 2018 and 2022. A total of 820 pregnant women were enrolled in this study; 410 pregnant women were diagnosed with HG (HG group) at 6–14 weeks of gestation, and 410 pregnant women were healthy controls (control group) in the same gestational weeks. Patients’ demographic and clinical characteristics and laboratory parameters were recorded. The hemoglobin, albumin, lymphocyte, and platelet (HALP) score, the modified-HALP (m-HALP) score, and the prognostic nutritional index (PNI) were calculated. **Results**: The HALP score (32.6 (IQR: 24.9–41.5) vs. 39.2 (IQR: 30.8–49.2), *p* < 0.001) and the PNI score (50 (IQR: 46.3–53.6) vs. 51.3 (IQR: 48.6–53.8), *p* < 0.001) were significantly lower in the HG group, whereas the m-HALP score was similar between the groups. The HALP score had an AUC of 0.625 (95% CI: 0.586–0.664), with the optimal cut-off value set at 35.8, resulting in a sensitivity of 59.7% and a specificity of 59.5% (*p* < 0.001). Similarly, the PNI score showed an AUC of 0.580 (95% CI: 0.541–0.619), and the optimal cut-off value was set at 50.6, resulting in a sensitivity of 54.9% and a specificity of 54.9% (*p* < 0.001). In regression analysis, lower HALP scores (OR: 0.906, 95% CI: 0.833–0.984, *p* = 0.019) and lower PNI scores (OR: 0.941, 95% CI: 0.891–0.995, *p* = 0.033) were significantly associated with HG, highlighting their potential as diagnostic markers. Additionally, a negative statistically significant correlation was observed between PNI scores and ketonuria (r = −0.073, *p* = 0.036). **Conclusions**: This study demonstrated a decrease in the HALP score and PNI score in cases of HG. However, the m-HALP score was similar in the HG and control groups.

## 1. Introduction

Nausea, with or without vomiting, is highly prevalent during the initial stages of pregnancy, to the extent that mild symptoms may be regarded as a typical physiological response in the first trimester. Approximately 70–80% of pregnancies are affected by nausea and vomiting [1]. Hyperemesis gravidarum (HG), a severe manifestation of nausea and vomiting during pregnancy, is characterized by significant weight loss, dehydration, electrolyte and acid–base disturbances, ketonuria, and nutritional deficiencies [2,3,4]. The prevalence of HG among pregnant women ranges from 0.8% to 3.2% [4,5,6]. Despite the unclear etiology of the condition, several theories have been proposed, including hormonal changes, immunologic factors, abnormal gastrointestinal motility, Helicobacter pylori infection, nutrient deficiencies, abnormalities in carbohydrate metabolism, endocrine disorders, alterations in lipid levels, changes in the autonomic nervous system, and genetic predisposition [7,8]. However, none of these theories have effectively explained the underlying causes of HG to date. Due to the lack of a definitive cause, current treatments for HG rely primarily on observation and are often insufficient.

Inflammation is thought to be one of the most important factors in the development of HG [9]. Recent investigations have shown that pregnant women with HG exhibit elevated levels of inflammatory markers during the first trimester compared to healthy pregnant women without HG. The research examining the correlation between inflammation and HG typically concentrates on the inflammatory biomarkers during the first trimester, as HG is predominantly observed during this period [10,11]. However, these count-based inflammatory markers are insufficient for disease diagnosis, prognosis, and follow-up, as results vary across studies. For this reason, indexes and scoring systems containing multiple markers have been developed. The hemoglobin, albumin, lymphocyte, and platelet (HALP) index is a novel score based on the combination of inflammation and nutritional status [12,13]. Similarly, the prognostic nutritional index (PNI) score, which is based on lymphocyte count and albumin levels, reflects both inflammation and nutritional status [14]. Both scores reveal systemic inflammation and nutritional status, and since they can be easily calculated, they can be used in diseases with inflammation.

Current research includes studies evaluating inflammatory markers in relation to HG, but these markers provide limited results because they are count-based [15,16]. HG is primarily diagnosed clinically, and identifying a reliable quantitative marker to predict its presence and severity could be critical for preventing poor outcomes. In addition, the relationship between ketonuria and hospitalization duration and the severity of inflammation has not been fully investigated. Therefore, in this study, we aimed to investigate the diagnostic value of inflammation indices between HG patients and healthy pregnancies and their relationship with hospitalization duration and ketonuria. To best our knowledge, this is the first study to investigate the relationship of the HALP score, modified HALP (m-HALP) score, and PNI with variables and the diagnostic value of the m-HALP score and PNI in patients with HG.

## 2. Materials and Methods

Clinical data from patients hospitalized with a diagnosis of HG in the Early Pregnancy Department of the Etlik Zubeyde Hanim Maternity, Teaching and Research Hospital between 2018 and 2022 were evaluated retrospectively. The control group consisted of healthy pregnant women with gestational ages matching those in the study group, who consistently attended our prenatal clinic. This study was conducted after ethical approval was obtained from the Health Sciences University Etlik Zubeyde Hanim Maternity, Teaching and Research Hospital Ethics Committee (Approval No: 20.12.2023–12). Informed consent was not necessary, due to the retrospective nature of this study, and it was waived by the Health Sciences University Etlik Zubeyde Hanim Maternity, Teaching and Research Hospital Ethics Committee. The principles of the Declaration of Helsinki were applied in this study. A total of 820 pregnant women were enrolled in this study; 410 pregnant women were diagnosed with HG (HG group) at 6–14 weeks of gestation, and 410 pregnant women were healthy controls (control group) in the same gestational weeks. Gestational age was determined using the first day of the last menstrual period and then confirmed by ultrasonography.

The hospital’s electronic medical system was used to collect the demographic and clinical information of all patients. The appropriate International Classification of Disease codes for HG were utilized for this purpose. Each patient’s age, body mass index (BMI), gestational age, gravidity, and parity were documented. BMI was calculated during the patients’ hospitalization. The criteria for HG were the following: experiencing severe vomiting at least three times per day, weight loss exceeding 5% of pre-pregnancy body weight, and having at least one positive result for ketonuria in the dipstick urine test. The participants were excluded if they had any inflammatory conditions and comorbidities such as hepatic, renal, or thyroid diseases; systemic or infectious diseases; eating disorders (anorexia, bulimia, etc.); high BMI (≥35 kg/m^2^); history of ovulation induction; anemia; gestational trophoblastic disease; hypertension; diabetes mellitus; gastrointestinal disorders; metabolic disorders; collagen vascular diseases; smoking; alcohol consumption; urinary tract infection; or pregnancy with multiple fetuses.

During hospitalization, routine blood and urine samples were collected as part of standard procedures. After being collected, all blood samples were tested on a daily basis using the same automated analyzer in the hematology laboratory of the hospital. The complete blood count (CBC) parameters, such as hemoglobin level, lymphocyte count, and platelet count, and biochemical parameters, such as albumin, alanine aminotransferase (ALT), aspartate aminotransferase (AST), and thyroid-stimulating hormone (TSH) values, were documented. Ketonuria was classified into four grades: 1+, 2+, 3+, and 4+. After that, immuno-nutritional indices, such as the HALP score, the m-HALP score, and the PNI score, were calculated with these values. The HALP score was calculated by using the hemoglobin (g/L) × albumin (g/L) × lymphocyte count (/L)/platelet count (/L) method. The m-HALP score was calculated by using the hemoglobin (g/L) × albumin (g/L) × lymphocyte count (/L) × platelet count (/L) method. The PNI score was calculated as 10 × serum albumin (g/dL) + 0.005 × total lymphocyte count (per mm^3^).

The patient was released from the hospital after experiencing an improvement in their ability to consume food orally.

### Statistical Analysis

The Statistical Package for the Social Sciences (SPSS) v26.0 software (IBM^®^ SPSS^®^ Statistics, Armonk, NY, USA) was used for the statistical analysis. Descriptive data are expressed as n, %, mean ± SD, and median (interquartile range (IQR)). The normality of the distributions was assessed using the Shapiro–Wilk test and histogram plots. Student’s t-test was used to compare normally distributed data, and these data are presented as the mean ± SD. The Mann–Whitney U test was used to analyze data that did not have a normal distribution, and these data are presented as the median (IQR). Categorical variables were compared using the Chi-square test. The relationship between the variables was investigated with Pearson’s correlation analysis. The area under the receiver operating characteristic (ROC) curve was used for cut-off values, sensitivity, and specificity. For multivariate analysis, factors identified by univariate analysis with *p* < 0.250 were then included in a binary logistic regression model to determine predictors of HG. The model’s fit was evaluated using the Hosmer–Lemeshow goodness-of-fit test. A type-I error level of 5% was considered the threshold for statistical significance. A *p*-value of <0.05 was considered statistically significant.

## 3. Results

The demographic, clinical, and laboratory characteristics and outcomes between the HG and control groups are shown in Table 1. No statistically significant differences were observed between the HG and control groups regarding maternal age, gravidity, parity, and gestational age (*p* > 0.05, for all). However, there were significant differences in BMI between groups; the HG group exhibited a lower BMI (27.2 ± 3.7 kg/m^2^) compared to the control group (29.7 ± 4.9 kg/m^2^) (*p* < 0.001). The HG group had higher levels of ALT (14 U/L vs. 13 U/L, *p* = 0.021), AST (18 U/L vs. 16.2 U/L, *p* < 0.001), and hemoglobin (13 g/dL vs. 12.7 g/dL, *p* = 0.001), and lower levels of TSH (0.75 mU/L vs. 1.47 mU/L, *p* < 0.001). Additionally, the HG group exhibited a higher platelet count and lower lymphocyte count (*p* < 0.05, for all). The HALP score [32.6 (24.9–41.5) vs. 39.2 (30.8–49.2), *p* < 0.001] and the PNI score [50 (46.3–53.6) vs. 51.3 (48.6–53.8), *p* < 0.001] were significantly lower in the HG group than in the control group, but the m-HALP score was statistically similar in both groups.

Table 2 illustrates the correlation between the PNI scores and various clinical–laboratory variables within the HG group, the control group, and the entire study cohort. Maternal age showed a positive correlation with PNI scores in the control group (r = 0.112, *p* = 0.023). Gestational age exhibited a significant negative correlation with PNI scores in the HG group (r = −0.142, *p* = 0.004) and the entire cohort (r = −0.119, *p* = 0.001). ALT displayed a positive correlation with PNI scores in the HG group (r = 0.098, *p* = 0.048). TSH demonstrated a negative correlation with PNI scores in the HG group (r = −0.103, *p* = 0.042). Hemoglobin, lymphocyte, and platelet counts exhibited positive correlations with PNI scores in both the HG group and the entire cohort (*p* < 0.05, for all). Similarly, albumin exhibited positive correlations with PNI scores in both the HG group and the entire cohort (*p* < 0.001).

Table 3 illustrates the correlation between the HALP scores and various clinical–laboratory variables within the HG group, the control group, and the entire study cohort. Gestational age displayed a significant negative correlation with HALP scores in the HG group (r = −0.113, *p* = 0.028), the control group (r = −0.119, *p* = 0.016), and the entire cohort (r = −0.071, *p* = 0.047). ALT displayed a positive correlation with HALP scores in the control group (r = 0.100, *p* = 0.043). TSH demonstrated a positive correlation with HALP scores in the entire group (r = 0.159, *p* < 0.001). Hemoglobin, lymphocyte count, and albumin were positively correlated with HALP scores in the HG group, the control group, and the entire cohort (*p* < 0.05, for all). Platelet counts exhibited negative correlations with HALP scores in both the HG group and the entire cohort (*p* < 0.05, for all).

Table 4 illustrates the correlation between the m-HALP scores and various clinical–laboratory variables within the HG group, the control group, and the entire study cohort. Maternal age demonstrated a significant negative correlation with m-HALP scores in the HG group (r =−0.122, *p* = 0.017) and the entire cohort (r = −0.090, *p* = 0.011). BMI displayed a positive correlation with m-HALP scores in the control group (r =0.234, *p* = 0.004) and the entire cohort (r = 0.180, *p* = 0.005). Gestational age exhibited a significant negative correlation with m-HALP scores in the HG group (r = −0.125, *p* = 0.015), the control group (r = −0.136, *p* = 0.006), and the entire cohort (r = −0.131, *p* < 0.001). ALT displayed a positive correlation with HALP scores in the control group (r = 0.098, *p* = 0.050). Hemoglobin, lymphocyte count, platelet count, and albumin were positively correlated with HALP scores in the HG group, the control group, and the entire cohort (*p* < 0.05, for all).

Table 5 shows the relationships between hospitalization duration, ketonuria, and various clinical–laboratory variables in HG patients. ALT (r = 0.115, *p* = 0.021) and platelet count (r = 0.120, *p* = 0.015) exhibited a positive correlation with hospitalization duration. A negative statistically significant correlation was observed between PNI scores and ketonuria (r = −0.073, *p* = 0.036).

**Figure 1:**
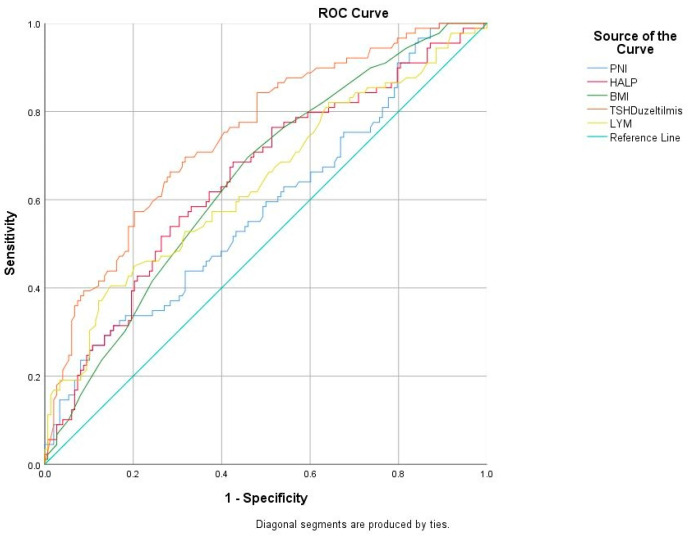
Receiver operating characteristic (ROC) curves to assess the usefulness of PNI score, HALP score, BMI, TSH, and lymphocyte count.

**Figure 2:**
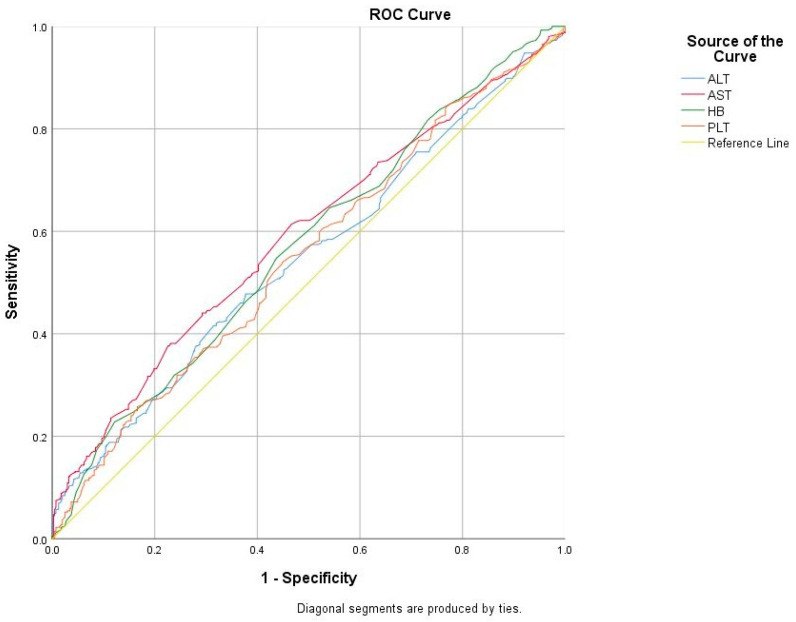
Receiver operating characteristic (ROC) curves to assess the usefulness of ALT, AST, hemoglobin, and platelet count.

To evaluate the usefulness of the PNI, HALP score, and other clinical–laboratory variables in distinguishing HG, ROC curves were calculated and are shown in Table 6. The HALP score had an AUC of 0.625 (95% CI: 0.586–0.664), with the optimal cut-off value set at 35.8, resulting in a sensitivity of 59.7% and a specificity of 59.5% (*p* < 0.001). Similarly, the PNI score showed an AUC of 0.580 (95% CI: 0.541–0.619), and the optimal cut-off value was set at 50.6, resulting in a sensitivity of 54.9% and a specificity of 54.9% (*p* < 0.001). Also, the AUC for BMI was 0.643 (cut-off: 27.5 kg/m^2^, 95% CI: 0.576–0.711, *p* < 0.001, sensitivity: 68.9%, specificity: 53.8%); for ALT, it was 0.547 (cut-off: 12 U/L, 95% CI: 0.507–0.586, *p* = 0.021, sensitivity: 57.3%, specificity: 49.1%); for AST, it was 0.588 (cut-off: 16.9 U/L, 95% CI: 0.549–0.627, *p* < 0.001, sensitivity: 61.1%, specificity: 53.3%); for TSH, it was 0.704 (cut-off: 1.15 mU/L, 95% CI: 0.667–0.740, *p* < 0.001, sensitivity: 66.2%, specificity: 65.1%); for hemoglobin, it was 0.569 (cut-off: 12.9 g/dL, 95% CI: 0.530–0.608, *p* = 0.001, sensitivity: 58.2%, specificity: 52.1%); for lymphocyte count, it was 0.637 (cut-off: 1.73 × 10^3^/μL, 95% CI: 0.599–0.675, *p* < 0.001, sensitivity: 60.7%, specificity: 60.2%); and for platelet count, it was 0.549 (cut-off: 259.5 × 10^3^/μL, 95% CI: 0.509–0.588, *p* = 0.016, sensitivity: 54.6%, specificity: 53.8%) (Figure 1 and Figure 2).

Regression analysis was performed to detect significant predictors of HG and are shown in Table 7. Lower BMI (OR: 0.850, 95% CI: 0.777–0.930, *p* < 0.001) and lower TSH levels (OR: 0.528, 95% CI: 0.344–0.809, *p* = 0.003) were significantly associated with higher odds of HG. Additionally, lower HALP scores (OR: 0.906, 95% CI: 0.833–0.984, *p* = 0.019) and lower PNI scores (OR: 0.941, 95% CI: 0.891–0.995, *p* = 0.033) were significantly associated with HG, highlighting their potential as diagnostic markers.

## 4. Discussion

HG is one of the severe health problems in early pregnancy. The pathogenesis of nausea and vomiting of pregnancy is unknown and likely multifactorial. Although many theories have been proposed, none of these theories are consistently associated with or highly predictive of HG. The precise function of inflammation in the pathogenesis of HG remains uncertain. However, it is possible that subclinical inflammation, which is linked to oxidative stress, could have a significant impact [10,17]. Although the link between HG development and inflammation is not fully understood, studies on inflammation markers in HG patients suggested strong connections between them [18,19]. Despite this, there is increasing but still insufficient interest in markers of inflammation as a diagnostic tool. So, our study conducted a comprehensive analysis of the HALP score, m-HALP score, and PNI in HG and healthy pregnancies, marking the first instance of such an analysis. In this retrospective case–control study, we demonstrated that HALP and PNI scores were lower in patients with HG than in healthy pregnant women, and this difference was statistically significant (*p* < 0.001, for all). We calculated ROC curves for the usefulness of the PNI, HALP score, and clinical–laboratory variables to distinguish HG from normal healthy pregnancy. The HALP score had an AUC of 0.625 (95% CI: 0.586–0.664), with the optimal cut-off value set at 35.8, resulting in a sensitivity of 59.7% and a specificity of 59.5% (*p* < 0.001). Similarly, the PNI score showed an AUC of 0.580 (95% CI: 0.541–0.619) and the optimal cut-off value was set at 50.6, resulting in a sensitivity of 54.9% and a specificity of 54.9% (*p* < 0.001). The m-HALP score could not significantly distinguish HG from the control group. In regression analysis to predict HG, lower HALP scores (OR: 0.906, 95% CI: 0.833–0.984, *p* = 0.019) and lower PNI scores (OR: 0.941, 95% CI: 0.891–0.995, *p* = 0.033) were significantly associated with HG, highlighting their potential as diagnostic markers.

During pregnancy, alterations in cellular and humoral immunity occur to safeguard the decidua and the fetus against the detrimental impacts of the maternal immune system. Once the maternal immune system has developed total immunological tolerance towards the fetus, trophoblasts aggressively infiltrate the decidua. Conversely, if sufficient maternal immunological tolerance cannot be attained, problems including miscarriage, preeclampsia, and HG may occur [20]. It is believed that inflammatory and immunological processes play a role in the pathophysiology of HG. Several studies have documented a robust correlation between the rise in specific cytokines, mediators, and acute phase reactants, and the occurrence of systemic inflammation and subsequent development of HG [9,10,18,21]. Nevertheless, this evidence is inadequate in determining whether inflammation is a causal factor or a consequence of HG [20,21]. Presently, it is crucial to evaluate the extent of maternal inflammation in order to anticipate unfavorable neonatal consequences. Although many inflammatory markers have been studied for the early diagnosis of HG, to best our knowledge, this is the first study to investigate the relationship of the HALP score, m-HALP score, and PNI with variables and the diagnostic value of the m-HALP score and PNI in patients with HG.

The HALP score, introduced in 2015 by Chen et al., combines measurements of immunological status (lymphocyte and platelet counts) and nutritional status (hemoglobin and albumin concentrations) that are regularly evaluated using standard laboratory tests [22]. Albumin, the predominant protein found in human serum, is a negative acute phase reactant commonly utilized as an indicator for nutritional status [23]. It can be influenced by clinical situations such as HG and liver disorders. In the meta-analysis conducted by Cabrerizo and colleagues, it was determined that serum albumin is a reliable indicator of nutritional status in older patients who are clinically stable [24]. Nevertheless, its effectiveness is constrained by its lack of precision and extended duration of activity [25]. The consensus is therefore that laboratory parameters, including albumin-derived indices, should be used alongside a thorough physical examination when assessing nutritional status [26]. During chronic inflammatory processes, excessive neutrophil activation can contribute to tissue damage, and lymphocyte count decreases due to increased apoptosis [27]. Platelets enable the initiation and modulation of immune functions by expressing several pro- and anti-inflammatory molecules [28]. In our study, BMI and lymphocyte count were found to be statistically significantly lower in the HG group, while hemoglobin level, platelet count, and AST and ALT levels were found to be high. Considering the role of these blood cells in inflammation separately, we thought their inclusion as a formula might be a better indicator of inflammation, so we evaluated HALP and m-HALP scores as a study parameter. We found the HALP score to be significantly lower in the HG group (*p* < 0.001). Hemoglobin, lymphocyte, and albumin showed a positive correlation with HALP and m-HALP scores in all groups (HG group, control group, and entire cohort). While platelets showed a positive correlation with m-HALP in all groups, there was a negative correlation with the HALP score in the HG group and the entire group. In agreement with our result, Bayram et al. found that in the first trimester, the HALP score was significantly lower in the HG group compared to the healthy pregnant group [29]. However, correlations between variables and the m-HALP score were not investigated in this study. Besides these, the HALP score was initially employed to forecast the prognosis of gastric cancer [22]. Subsequently, it was employed to forecast the likely course of prostate cancer, bladder cancer, lung cancer, colorectal cancer, and individuals with ischemic stroke [30,31,32,33,34]. Studies have shown that a low HALP score is associated with a poor prognosis. Kocaoglu et al. examined the m-HALP score and, for the first time in the literature, the m-HALP score in patients with acute heart failure (AHF) [35]. They examined the relationship between the m-HALP score and the classic HALP score with mortality in patients with AHF and found that the m-HALP score was a significant predictor of 3-month mortality in patients with AHF, while the classic HALP score was not significant for 1-week and 3-month mortality. In contrast to this, in our study, the classical HALP score was shown to be more meaningful than the m-HALP score for HG.

The PNI score, which is calculated based on the serum albumin concentration and total lymphocyte count in the peripheral blood, can reflect the immune–nutritional condition [36]. The PNI was firstly developed by Onodera et al. as a risk marker for post-operative complications following gastrointestinal surgery [37]. In some studies, a low PNI has been shown to be a negative prognostic factor in cancers of the ovary [38], cervix [39], lung [40], colon [41], and pancreas [42]. We observed that the PNI score was lower in the HG group (*p* < 0.001). In our study, a positive correlation was observed between hemoglobin, platelet, lymphocyte, and albumin levels with the PNI score in both the HG group and the entire cohort. As is known, while hemoglobin and albumin levels indicate the nutritional status of the body, lymphocytes and platelets are associated with immune status [23,25,26,28]. These findings underscore the interplay between immune–nutritional status and HG.

Urine typically contains acetoacetate and beta-hydroxybutyric acid as ketone bodies when lipids are used for energy [43]. Ketonuria is utilized as a criterion to comprehend the metabolic repercussions and clinical prognosis of patients with HG, while its correlation with the severity of HG remains uncertain. Only a few studies examine the potential correlation between the severity of HG and various factors such as the duration of hospital stay, the likelihood of readmission, metabolic and biochemical indicators, hematological parameters, clinical characteristics, and inflammatory markers [44]. In our study, we found a statistically significant and negative correlation only between ketonuria and the PNI. The HALP score and m-HALP score were not significantly correlated with ketonuria. This showed us that as nutrition deteriorates in HG patients, ketonuria increases and the PNI decreases.

In our study, we did not find a statistically significant difference between the control group with a healthy pregnancy and the HG group in terms of gravidity, parity, and maternal age. The literature is controversial on this issue, with some studies finding younger, primigravida individuals to be significantly more likely to have HG [45], as well as studies showing no significant difference [46]. In addition, in our study, while a positive correlation was observed between maternal age and the PNI score in the control group, a significant negative correlation was observed between the m-HALP score and maternal age in the HG group and the entire cohort.

The most important strengths of this study are its strict patient selection criteria, relatively high number of patients, and the fact that the PNI and m-HALP scores were studied for HG for the first time. There are limitations in this study. Initially, our study is retrospective in nature. The weight change data throughout pregnancy were inaccessible in the dataset. The population selection may exhibit bias due to its inclusion of patients exclusively monitored by a single center and treated using a uniform technique. Moreover, these inflammatory–nutritional parameters for diagnosing HG and predicting its severity are limited by relatively low sensitivity and specificity. Of course, the mechanism of HG and the course of pregnancy are complex, so it may be assertive to claim that we can predict HG in pregnant women using only some blood cell parameters. The HALP score and PNI may be more practical and effective when used in conjunction with other risk factors and biomarkers in these vulnerable populations.

## 5. Conclusions

In conclusion, this study demonstrated a decrease in the PNI score and HALP score in cases of HG. However, the m-HALP score could not differentiate HG from the control group. This suggests that immunological variables play a significant role in the development of HG. Further research is necessary to investigate and validate these biomarkers related to prognosis, inflammation, immune response, and nutritional status in individuals with HG.

## Figures and Tables

**Table 1:** Demographic, clinical, and laboratory characteristics and outcomes between the HG and control groups.

	HG Group n: 410	Control Group n: 410	*p*-Value
Maternal age (year)	28 ± 6	28 ± 6	0.539
Gravidity	2 ± 1	2 ± 1	0.778
Parity	1 ± 1	1 ± 1	0.131
BMI (kg/m^2^)	27.2 ± 3.7	29.7 ± 4.9	<0.001
Gestational age (week)	9.4 (7.4–12)	9.1 (7.4–11.2)	0.093
ALT (U/L)	14 (11–21.6)	13 (10–18)	0.021
AST (U/L)	18 (15–22.9)	16.2 (14.7–19)	<0.001
TSH (mU/L)	0.75 (0.28–1.40)	1.47 (0.89–2.15)	<0.001
Hemoglobin (g/dL)	13 ± 1.1	12.7 ± 1.2	0.001
Lymphocyte (×10^3^/μL)	1.60 (1.29–2.01)	1.88 (1.54–2.24)	<0.001
Platelet (×10^3^/μL)	271.5 ± 63.9	261.6 ± 60.7	0.016
Albumin (g/dL)	41.8 (39.4–44.7)	41.6 (39.4–44.9)	0.468
Fibrinogen (mg/dL)	338.5 (304.5–407.5)	350 (300–397)	0.446
PNI score	50 (46.3–53.6)	51.3 (48.6–53.8)	<0.001
HALP score	32.6 (24.9–41.5)	39.2 (30.8–49.2)	<0.001
m-HALP score	2,395,973.58 (1,729,770.09–3,207,963.61)	2,447,363.54 (1,923,999.89–3,274,174.10)	0.065
Ketonuria	1.93 ± 1.79	-	N/A
Antiemetic drug use	410 (100%)	-	N/A
Hospitalization duration (day)	2 (1–3)	-	N/A

Abbreviations: BMI: body mass index, ALT: alanine aminotransferase, AST: aspartate aminotransferase, TSH: thyroid-stimulating hormone, PNI: prognostic nutritional index, HALP: hemoglobin, albumin, lymphocyte, and platelet score, m-HALP: modified hemoglobin, albumin, lymphocyte, and platelet score. Data are shown as mean ± SD, median (IQR), or n, %.

**Table 2:** The relationship between PNI scores and clinical–laboratory variables.

	HG Group		Control Group		Entire Cohort	
	r	*p*-Value	r	*p*-Value	r	*p*-Value
Maternal age	−0.036	0.467	0.112	0.023	−0.043	0.218
Gravidity	−0.024	0.669	0.028	0.591	−0.038	0.309
Parity	0.002	0.964	0.033	0.523	−0.036	0.328
BMI	0.050	0.616	0.069	0.161	0.081	0.195
Gestational age	−0.142	0.004	0.060	0.278	−0.119	0.001
ALT	0.098	0.048	0.033	0.552	0.068	0.054
AST	0.086	0.083	−0.160	0.107	0.048	0.172
TSH	−0.103	0.042	0.025	0.619	0.024	0.508
Hemoglobin	0.175	<0.001	−0.013	0.796	0.149	<0.001
Lymphocyte	0.243	<0.001	−0.041	0.413	0.307	<0.001
Platelet	0.096	0.050	−0.024	0.643	0.069	0.049
Albumin	0.782	<0.001	0.041	0.410	0.796	<0.001
Fibrinogen	−0.083	0.385	0.012	0.804	−0.084	0.148

Abbreviations: BMI: body mass index, ALT: alanine aminotransferase, AST: aspartate aminotransferase, TSH: thyroid-stimulating hormone, PNI: prognostic nutritional index.

**Table 3:** The relationship between HALP scores and clinical–laboratory variables.

	HG Group		Control Group		Entire Cohort	
	r	*p*-Value	r	*p*-Value	r	*p*-Value
Maternal age	−0.077	0.135	−0.085	0.086	−0.049	0.170
Gravidity	−0.034	0.556	−0.055	0.269	−0.037	0.332
Parity	−0.030	0.595	−0.076	0.129	−0.050	0.182
BMI	−0.067	0.525	−0.003	0.966	0.107	0.097
Gestational age	−0.113	0.028	−0.119	0.016	−0.071	0.047
ALT	0.009	0.860	0.100	0.043	−0.030	0.402
AST	0.036	0.481	0.045	0.367	−0.042	0.239
TSH	0.090	0.089	0.090	0.077	0.159	<0.001
Hemoglobin	0.318	<0.001	0.230	<0.001	0.386	<0.001
Lymphocyte	0.700	<0.001	0.419	<0.001	0.668	<0.001
Platelet	−0.420	<0.001	0.080	0.108	−0.438	<0.001
Albumin	0.158	0.002	0.822	<0.001	0.219	<0.001
Fibrinogen	0.126	0.199	−0.100	0.174	−0.002	0.979

Abbreviations: BMI: body mass index, ALT: alanine aminotransferase, AST: aspartate aminotransferase, TSH: thyroid-stimulating hormone, HALP: hemoglobin, albumin, lymphocyte, and platelet score.

**Table 4:** The relationship between m-HALP scores and clinical–laboratory variables.

	HG Group		Control Group		Entire Cohort	
	r	*p*-Value	r	*p*-Value	r	*p*-Value
Maternal age	−0.122	0.017	−0.067	0.178	−0.090	0.011
Gravidity	−0.094	0.099	−0.039	0.445	−0.065	0.083
Parity	−0.080	0.162	−0.041	0.412	−0.064	0.090
BMI	0.045	0.668	0.234	0.004	0.180	0.005
Gestational age	−0.125	0.015	−0.136	0.006	−0.131	<0.001
ALT (U/L)	0.085	0.097	0.098	0.050	0.067	0.060
AST (U/L)	0.063	0.219	−0.049	0.322	0.008	0.824
TSH	0.079	0.132	0.011	0.825	0.056	0.127
Hemoglobin	0.328	<0.001	0.325	<0.001	0.311	<0.001
Lymphocyte	0.780	<0.001	0.805	<0.001	0.786	<0.001
Platelet	0.646	<0.001	0.618	<0.001	0.621	<0.001
Albumin	0.218	<0.001	0.240	<0.001	0.226	<0.001
Fibrinogen	0.079	0.421	0.142	0.054	0.313	0.055

Abbreviations: BMI: body mass index, ALT: alanine aminotransferase, AST: aspartate aminotransferase, TSH: thyroid-stimulating hormone, m-HALP: modified hemoglobin, albumin, lymphocyte, and platelet score.

**Table 5:** The relationship between hospitalization duration, ketonuria, and clinical–laboratory variables in HG patients.

	Hospitalization Duration		Ketonuria	
	r	*p*-Value	r	*p*-Value
PNI score	0.057	0.253	−0.073	0.036
HALP score	−0.087	0.092	0.028	0.591
m-HALP score	0.005	0.921	0.033	0.523
Maternal age	0.053	0.284	0.069	0.161
Gravidity	0.052	0.343	0.060	0.278
Parity	0.070	0.207	0.033	0.552
BMI	0.005	0.964	−0.160	0.107
Gestational age	−0.004	0.932	0.025	0.619
ALT (U/L)	0.115	0.021	−0.013	0.796
AST (U/L)	0.090	0.071	−0.041	0.413
TSH	−0.046	0.369	−0.024	0.643
Hemoglobin	−0.045	0.360	0.041	0.410
Lymphocyte	−0.037	0.454	0.012	0.804
Platelet	0.120	0.015	0.034	0.498
Albumin	−0.053	0.302	−0.041	0.420
Fibrinogen	−0.004	0.967	0.056	0.557

Abbreviations: PNI: prognostic nutritional index, HALP: hemoglobin, albumin, lymphocyte, and platelet score, m-HALP: modified hemoglobin, albumin, lymphocyte, and platelet score, BMI: body mass index, ALT: alanine aminotransferase, AST: aspartate aminotransferase, TSH: thyroid-stimulating hormone.

**Table 6:** Receiver operating characteristic (ROC) curves to evaluate the usefulness of PNI, HALP score, and clinical–laboratory variables in differentiating HG.

	AUC	95% CI	*p*-Value	Cut-Off Value	Sensitivity (%)	Specificity (%)
PNI score	0.580	0.541–0.619	<0.001	50.6	54.9	54.9
HALP score	0.625	0.586–0.664	<0.001	35.8	59.7	59.5
BMI (kg/m^2^)	0.643	0.576–0.711	<0.001	27.5	68.9	53.8
ALT (U/L)	0.547	0.507–0.586	0.021	12	57.3	49.1
AST (U/L)	0.588	0.549–0.627	<0.001	16.9	61.1	53.3
TSH (mU/L)	0.704	0.667–0.740	<0.001	1.15	66.2	65.1
Hemoglobin (g/dL)	0.569	0.530–0.608	0.001	12.9	58.2	52.1
Lymphocyte (×10^3^/μL)	0.637	0.599–0.675	<0.001	1.73	60.7	60.2
Platelet (×10^3^/μL)	0.549	0.509–0.588	0.016	259.5	54.6	53.8

Abbreviations: AUC: Area under the curve, CI: confidence interval, PNI: prognostic nutritional index, HALP: hemoglobin, albumin, lymphocyte, and platelet score, BMI: body mass index, ALT: alanine aminotransferase, AST: aspartate aminotransferase, TSH: thyroid-stimulating hormone.

**Table 7:** Regression analysis to predict HG.

	OR	95% CI	*p*-Value
Parity	1.374	0.997–1.894	0.052
BMI (kg/m^2^)	0.850	0.777–0.930	<0.001
Gestational age (week)	0.979	0.834–1.150	0.796
ALT (U/L)	1.002	0.977–1.027	0.889
AST (U/L)	1.045	0.985–1.108	0.142
TSH (mU/L)	0.528	0.344–0.809	0.003
Hemoglobin (g/dL)	1.490	0.939–2.363	0.090
Lymphocyte (×10^3^/μL)	0.459	0.069–3.048	0.420
Platelet (×10^3^/μL)	0.994	0.978–1.012	0.524
PNI score	0.941	0.891–0.995	0.033
HALP score	0.906	0.833–0.984	0.019
m-HALP score	1.000	1.000–1.053	0.270

Abbreviations: OR: odds ratio, CI: confidence interval, BMI: body mass index, ALT: alanine aminotransferase, AST: aspartate aminotransferase, TSH: thyroid-stimulating hormone, PNI: prognostic nutritional index, HALP: hemoglobin, albumin, lymphocyte, and platelet score, m-HALP: modified hemoglobin, albumin, lymphocyte, and platelet score.

## Data Availability

On reasonable request, the corresponding author will provide the information supporting this study’s conclusions.
